# Associations between food group intake and serum levels of selenium and other essential and toxic trace elements in adults

**DOI:** 10.1007/s00394-026-03922-y

**Published:** 2026-02-28

**Authors:** Inés Rivas, Marta Miranda, Carlos Herrero-Latorre, Rafael Monte-Secades, Marta López-Alonso

**Affiliations:** 1 Escola Universitaria de Enfermería de Lugo, Servizo Galego de Saúde, Universidade de Santiago de Compostela, 27002 Lugo, Spain; 2 Hospital Universitario Lucus Augusti, 27003 Lugo, Spain; 3 Departamento de Anatomía, Produción Animal e Ciencias Clínicas Veterinarias, Facultade de Veterinaria, Universidade de Santiago de Compostela, Campus Terra, 27002 Lugo, Spain; 4 Departamento de Química Analítica, Nutrición e Bromatoloxía, Facultade de Ciencias, Instituto de Investigación do Medio Acuático para unha Saúde Global (iARCUS), Universidade de Santiago de Compostela, Campus Terra, Lugo, 27002 Spain; 5 Servicio de Medicina Interna. Hospital Universitario Lucus Augusti, Lugo, 27003 Spain; 6 Departamento de Patoloxía Animal, Facultade de Veterinaria, Universidade de Santiago de Compostela, Campus Terra, Lugo, 27002 Spain

**Keywords:** Selenium, Essential elements, Toxic elements, Serum, Food-intake

## Abstract

**Purpose:**

To examine how the habitual consumption of major food groups is related to serum concentrations of essential and toxic trace elements in adults and to identify key dietary predictors of adequate status.

**Methods:**

In this cross-sectional analysis, 465 healthy adults (Galicia, Spain; 2020–2022) completed a validated semi-quantitative food frequency questionnaire; foods were categorised in 13 groups. Fasting serum concentrations of 14 trace elements were measured by ICP-MS. Group differences across element tertiles (clinical categories for selenium) were compared using the Kruskal–Wallis test. Random Forest (RF) models were constructed to evaluate multivariate dietary predictors for each element. Age- and sex-adjusted logistic regression were used to identify food group predictors of adequate (> 90 µg/L) vs. non-adequate selenium.

**Results:**

Seafood intake was positively associated with serum selenium concentrations and strongly tracked serum arsenic and mercury concentrations. Dairy, fruit and meat were also included in the RF models. Seafood ranked among the top three RF predictors for 13/14 elements; dairy ranked in all models. Logistic regression indicated higher odds of adequate selenium with greater seafood intake (OR 1.009; 95% CI 1.003–1.015; *p* = 0.007) and inverse associations with oil (OR 0.947; *p* = 0.018) and grains (OR 0.992; *p* = 0.036); positive trends in legume and nut consumption were identified. Individuals with adequate selenium reported consumption of ~ 4 fish servings/week.

**Conclusion:**

Integrating dietary and biomarker data revealed selenium to be the most vulnerable micronutrient. Promoting regular consumption of fish (and possibly nuts) while moderating high-oil/high-grain intake may enhance selenium without exceeding toxic metal reference limits.

## Introduction

Essential trace elements, such as zinc, iron, selenium and iodine, are required in relatively small amounts compared with macronutrients and are tightly regulated by homeostatic mechanisms [1]. They play a critical role in maintaining fundamental biological functions such as immune defense, antioxidant protection, thyroid regulation and cognitive development [2, 3]. However, imbalances in levels can lead to health problems: deficiencies are common and often subclinical, while excessive accumulation may also cause harm [4, 5]. By contrast, non-essential trace elements such as mercury, lead, arsenic and cadmium do not have any known physiological functions and are inherently toxic [2]. Even at low concentrations, they can accumulate in tissues and cause damage [6]. Together, both insufficient intake of essential elements and exposure to toxic elements greatly contribute to the global burden of disease, particularly among vulnerable populations such as children, pregnant women and the elderly [7, 8].

Nonetheless, in high-income countries, deficiencies of certain essential elements, particularly zinc, iodine and selenium, may still occur despite adequate caloric intake, especially in diets low in animal-source or nutrient-dense foods [9–12]. At the same time, chronic low-level exposure to toxic elements remains of concern due to environmental contamination and accumulation in specific food chains, notably seafood and rice. Institutions such as the World Health Organization (WHO), the Food and Agriculture Organization (FAO) and the European Food Safety Authority (EFSA) have prioritized the assessment of both nutritional adequacy and toxic risk related to trace elements in food systems [13–15].

Estimating micronutrient status at the population level often involves the use of dietary intake data derived from self-reported assessment tools, such as food frequency questionnaires, in combination with food composition tables. However, this approach is subject to substantial uncertainty due to reporting bias in dietary surveys and variability in food composition data. It should be noted that population reference values established by international agencies do take into account absorption, bioavailability and metabolic considerations at the population level. Nevertheless, these reference values are necessarily based on average assumptions and cannot fully capture interindividual variability related to age, physiological status, health conditions, dietary matrices or nutrient–nutrient interactions. Moreover, these approaches have important limitations, as they do not account for interindividual variation in absorption and metabolism, interactions between nutrients, food preparation methods or the actual bioavailability of micronutrients in complex dietary matrices [2]. These limitations are particularly important in relation to elements with narrow physiological ranges or for which circulating levels are tightly regulated and may not accurately reflect body stores. As a result, biomarker-based approaches, involving determination of serum or plasma levels, are increasingly recognized as valuable tools in nutritional epidemiology and public health research [16]. For many trace elements, serum concentrations reflect recent intake and absorption and can provide early warning signs of deficiency or excessive exposure [2].

In this context, selenium has emerged as an element of particular interest in nutritional epidemiology. Its relatively narrow range between inadequate and excessive intake, high inter-individual variability in intake and strong dependence on local soil concentrations make dietary intake estimates particularly unreliable [17, 18]. Moreover, recent findings from our cohort study conducted in north-western Spain have shown that more than half of the adult population has suboptimal serum selenium levels, underscoring a regional nutritional deficit that may compromise immune function and antioxidant capacity [19]. These findings reinforce the importance of biomarker-based monitoring and highlight the need to identify modifiable dietary factors contributing to selenium status.

Understanding the dietary determinants of selenium status is particularly important given the established role of this element in chronic disease prevention, including its involvement in immune regulation, inflammation control and oxidative stress mitigation [20]. However, selenium is not the only element of concern. The broader impact of trace elements (both essential and toxic) on long-term health outcomes is increasingly recognized [2]; however, comprehensive studies exploring their relationship with habitual dietary patterns remain scarce. Most existing research is limited to a small number of elements or relied on indirect intake estimates derived from dietary questionnaires and food composition tables, failing to capture the complexity of real-world dietary patterns.

To address this research gap, the present study aimed to evaluate the associations between food group intake and serum concentrations of a broad panel of essential and toxic trace elements in the previously characterized adult cohort in north-western Spain. Particular attention was given to selenium, for which a separate logistic regression model was constructed to identify the most relevant dietary predictors of adequate serum status. By combining univariate and multivariate statistical analyses, including Random Forest modelling, this study contributes to the emerging field of precision nutrition and provides evidence-based insights to guide targeted dietary strategies for improving micronutrient status.

## Materials and methods

### Study population

Participants were adults aged 18–79 years residing in Galicia (NW Spain), recruited between 2020 and 2022 as part of a larger population-based study (*n* = 501) previously described by Rivas et al. [19]. For the present analysis, 465 individuals with complete dietary and serum trace element data were included.

Eligibility criteria required participants to be in apparent good general health. Exclusion criteria included active oncological disease, pregnancy, chronic inflammatory, hepatic or renal diseases, recent acute infections, diagnosed nutritional disorders, and the use of pharmacological or nutritional treatments (including mineral supplementation) that could interfere with trace element metabolism. All participants provided written informed consent prior to inclusion, and the study protocol was approved by Ethics Committee of Galicia, Spain (code 2022/034).

Participants were recruited on a voluntary basis, following a convenience-based sampling approach. Although recruitment was not formally stratified, efforts were made to include individuals of both sexes, a wide age range and different geographic areas within Galicia. As commonly observed in voluntary health studies, participation was higher among women, and a perfectly balanced distribution across sex and age groups could not be fully achieved.

### Data collection and analysis

Fasting venous blood samples were collected using trace element–free serum tubes, with approximately 6 mL obtained from each participant. Samples were centrifuged immediately (3,500 rpm for 5 min) to separate serum, which was aliquoted and stored at − 80 °C until analysis. Serum concentrations of essential and toxic trace elements (arsenic, cadmium, cobalt, chromium, copper, iodine, iron, manganese, mercury, molybdenum, nickel, lead, selenium and zinc) were measured by inductively coupled plasma mass spectrometry (ICP-MS) (Agilent 7900×ICP-MS system; Agilent Technologies, Tokyo, Japan) in the Research Infrastructures Unit of the University of Santiago de Compostela (Lugo, Spain). For many trace elements, serum or whole-blood concentrations are widely used biomarkers of dietary intake and exposure in epidemiological studies, although the time window reflected varies across elements [21, 22]. All samples were analyzed in triplicate. Analytical quality control included procedural blanks, certified reference materials for human serum, spiked samples and internal standards. Limits of detection were calculated as three times the standard deviation of blank measurements, and intra- and inter-assay precision were routinely assessed. Full analytical protocols are described in detail elsewhere [19].

Dietary intake data were obtained using a semi-quantitative food frequency questionnaire (FFQ) previously validated for the Spanish population [23] and administered by trained personnel. The FFQ was designed to assess habitual dietary intake over the previous 12 months and included approximately 154 individual food items representative of the habitual Spanish diet. Participants reported the usual frequency of consumption (daily, weekly, monthly) and portion size for each food item. Reported frequencies were converted into average daily intake, and portion sizes were transformed into grams per day using standard portion definitions according to Velho et al. [24] and Carbajal [25] . Based on this detailed input, food items were subsequently grouped into 13 food categories (fruit, vegetables, legumes, grains, nuts, dairy, eggs, meat, seafood, sweets, processed food, oil and wine) consistent with the original structure of the FFQ and following the methodology described in Rivas et al. [26]. Questionnaires presenting implausible or internally inconsistent dietary information were excluded to ensure data quality. Additional sociodemographic and lifestyle data were also collected through the same structured interview.

### Statistical analysis

A descriptive analysis of serum trace element concentrations and dietary intake patterns was conducted. Associations between dietary variables and serum levels were first explored using non-parametric Kruskal–Wallis tests [27], comparing food group consumption across serum concentration tertiles (T1–T3) for each element. For selenium, clinically defined categories were used instead of tertiles, based on thresholds proposed by Rivas et al. [19]: deficient (< 60 µg/L), suboptimal (60–90 µg/L), and adequate (> 90 µg/L).To complement these univariate analyses and account for potential nonlinearities and variable interactions, Random Forest (RF) classification models [28] were used. This multivariate, non-parametric approach is particularly suited to complex nutritional datasets, as it can capture both nonlinear effects and inter-variable dependencies. Serum trace element concentrations were categorized into tertiles (T1–T3) and used as the outcome variable, with separate RF models developed for each trace element. Only dietary variables were included as predictors, excluding demographic or clinical covariates to isolate dietary effects. Variable importance was assessed using the mean decrease in Gini index, and model accuracy was evaluated through classification metrics and out-of-bag (OOB) error estimates.

To further investigate the dietary determinants of selenium status, a logistic regression model was applied [29, 30], using a binary outcome variable (0 = deficient and suboptimal selenium level, 1 = adequate selenium level). Predictor variables included all 13 food groups, aiming to identify those most strongly associated with adequate serum selenium status. For clarity of presentation, only food groups showing statistically significant associations, together with those showing a trend towards statistical significance, are reported Parameters were estimated using maximum likelihood, and odds ratios (ORs) with 95% confidence intervals were reported. Logistic regression was selected due to its interpretability and robust performance with categorical outcomes in epidemiological research.

All statistical analyses were performed using R software and Statgraphics Centurion XIX. A nominal *p*-value < 0.05 was used as a reference threshold, although univariate analyses were interpreted in an exploratory manner as described above.

## Results

### Univariate analysis by serum tertiles

Descriptive analysis and Kruskal–Wallis tests revealed associations between food group intake and serum concentrations of multiple essential and toxic trace elements. Table 1 presents median intakes for each food group across serum concentration tertiles for all elements analyzed (clinical categories for selenium), along with *p*-values indicating differences across serum concentration categories.

**Table 1:**
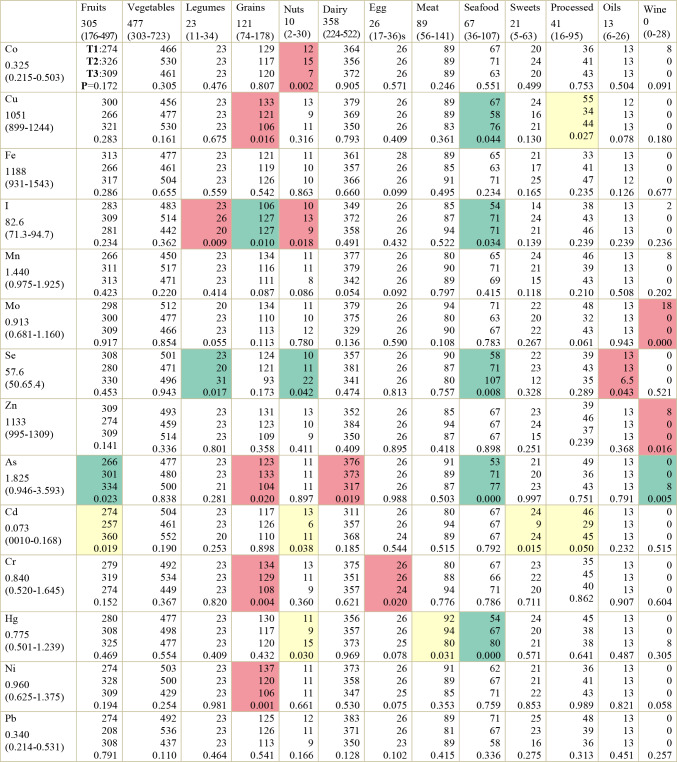
Associations between food group consumption and serum trace element levels based on univariate analysis (Kruskal–Wallis test)

Overall values for the total study population are shown below each serum trace element and food group as median (interquartile range, 25th–75th percentile). Values presented in the table correspond to median food group intake within each tertile (T1, T2 and T3) of serum trace element concentrations. For selenium, clinical categories were used instead of tertiles. The shading in cells indicate the direction of the observed pattern, where green denotes a positive association (higher intake in higher tertiles), red a negative association (higher intake in lower tertiles) and yellow an undefined or non-linear pattern

**Fig. 1:**
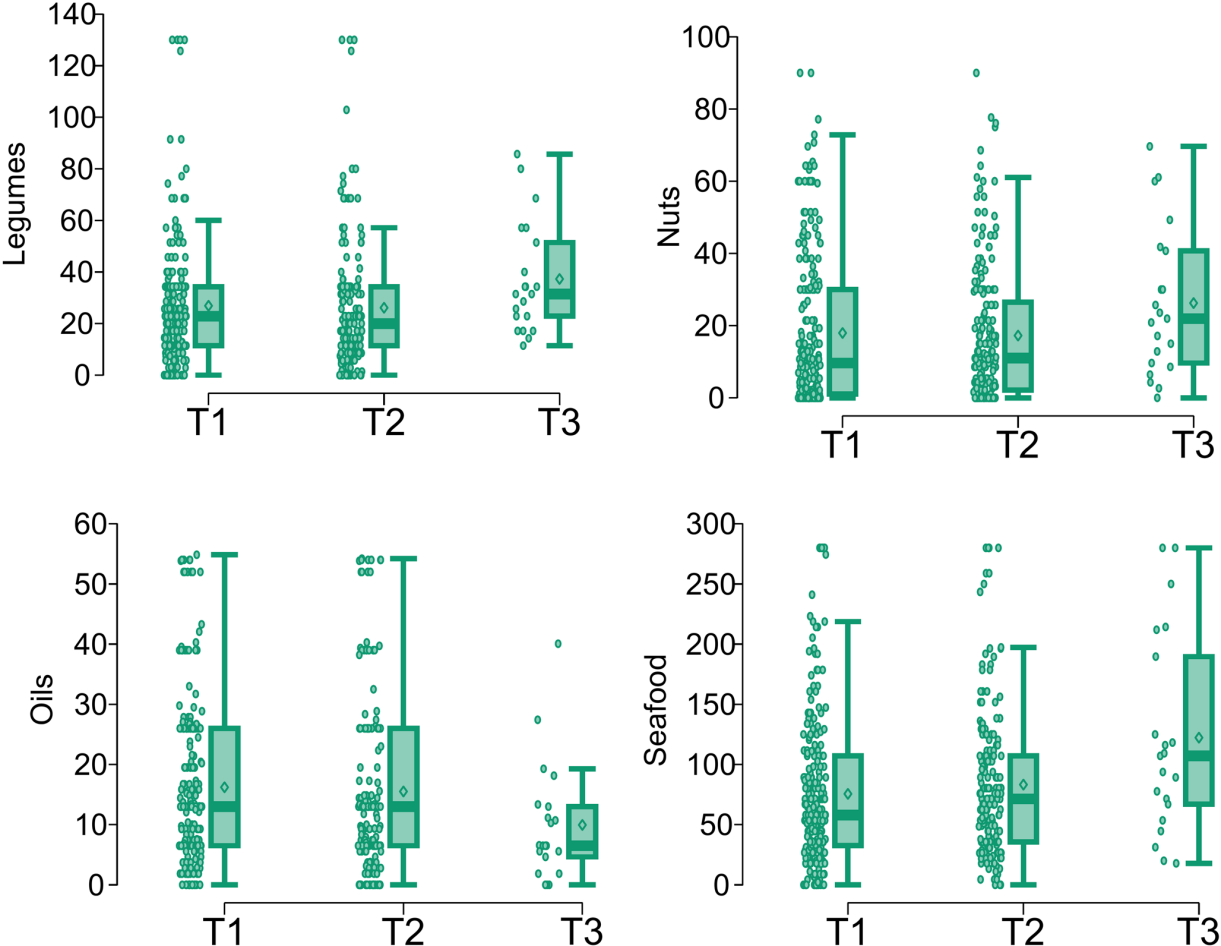
Distribution of serum selenium (Se) concentrations in relation to food group categories showing significant differences across clinical categories (T1: Deficient [< 60 µg/L], T2: Suboptimal [60–90 µg/L], T3: Optimal [> 90 µg/L])

Among essential trace elements, selenium showed the most consistent dietary associations in the univariate analysis, with higher serum levels corresponding to greater intake of legumes, nuts and seafood, and lower intake of oil. These trends are illustrated in Fig. 1, in which box-and-whisker plots represent food intake across selenium categories. Copper levels were positively associated with seafood consumption and negatively with processed and grain-based foods. Zinc and molybdenum levels exhibited inverse relationships with wine consumption, as did cobalt levels with nuts. Iodine levels were positively associated with seafood and grain consumption, and inversely associated with legume and nut consumption.

Among toxic elements, arsenic and mercury stood out with clear positive associations with seafood intake. Arsenic levels were also positively associated with fruit and wine consumption and inversely related to grains and dairy. Mercury levels were inversely associated with meat intake. Levels of both nickel and chromium were inversely associated with grain consumption, and chromium levels were also inversely associated with egg consumption. Cadmium accumulation exhibited a non-linear distribution: the intake of fruit, nuts, sweets and processed foods was lowest in the second tertile, while similar levels were observed in the first and third tertiles.

**Fig. 2:**
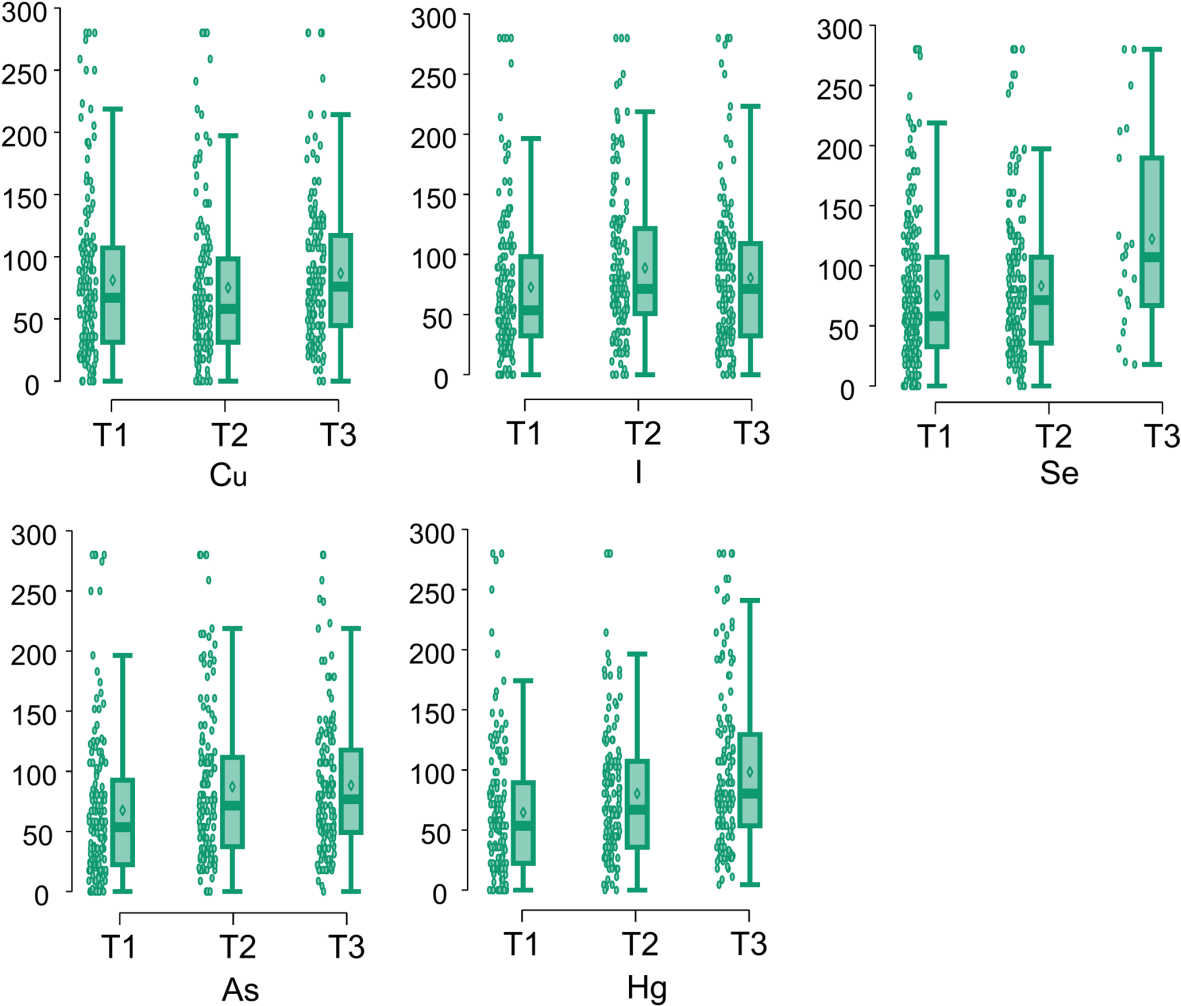
Distribution of seafood consumption in relation to copper (Cu), iodine (I), selenium (Se, clinical categories), arsenic (As) and mercury (Hg) serum levels (tertiles), highlighting its strong association with both essential and toxic elements

Among all food groups, seafood emerged as the most influential, showing consistent associations with both essential and toxic elements across the univariate analysis. As illustrated in Fig. 2, higher consumption of seafood was associated with higher levels of selenium, arsenic and mercury, reflecting its dual nutritional and toxicological impact in the diet.

Nevertheless, the univariate associations do not account for potential interactions, collinearity or nonlinear effects among dietary factors. To address these limitations and better understand the combined dietary contribution to trace element status, we used Random Forest classification models.

## Multivariate analysis using random forest

**Fig. 3:**
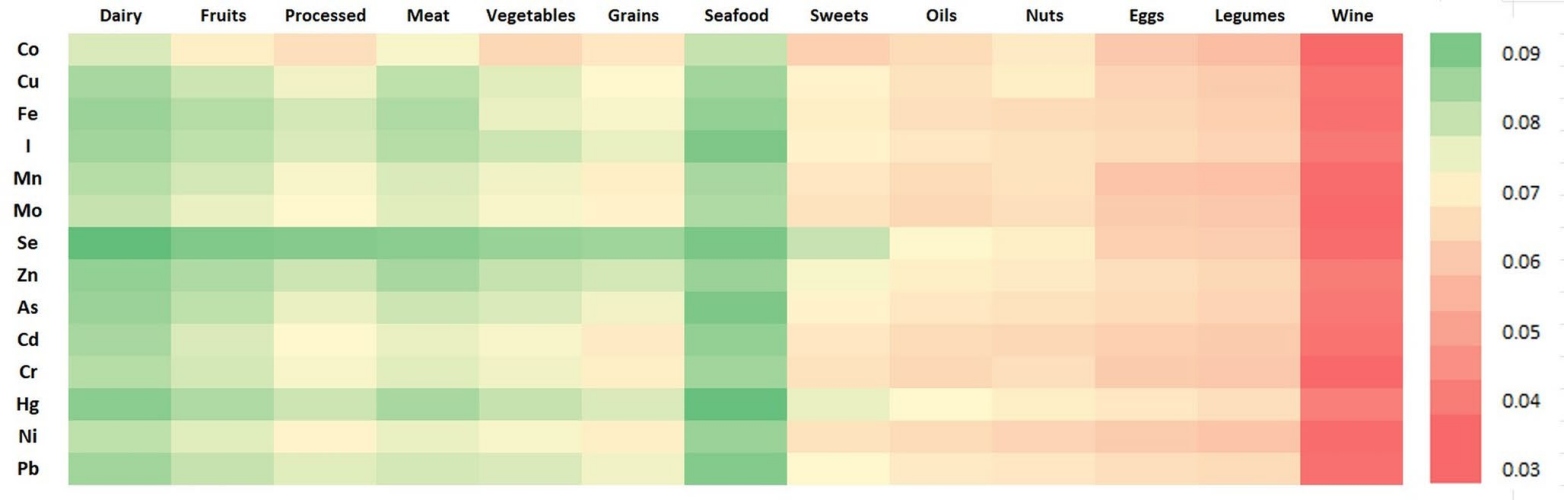
Variable importance plot from Random Forest models across all elements, showing the top predictive food groups for each trace element. Importance is based on the mean decrease in Gini index

The Random Forest analysis identified the relative importance of dietary food groups in relation to serum trace element categories. Variable importance plots (Fig. 3) summarize the contribution of each food group across the different trace elements analyzed.

**Fig. 4:**
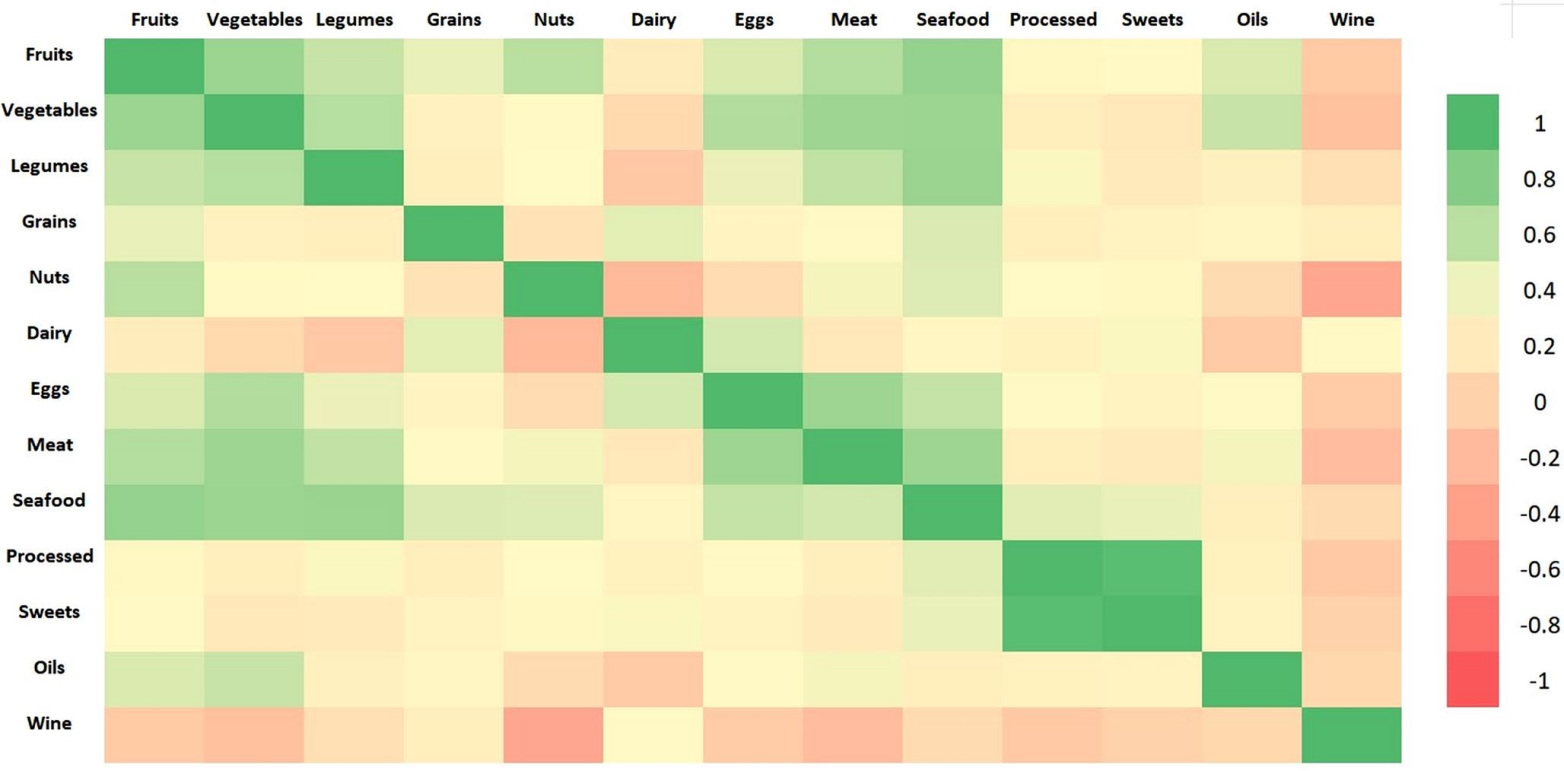
Heatmap showing co-consumption patterns among food groups based on Spearman correlations. Different colours represent the strength and direction of association, from negative (red) to positive (green), with darker shades indicating stronger correlations

Seafood and dairy products emerged as the most consistent predictors across elements. Seafood appeared among the top three predictors in 13 of 14 models, supporting its dual role as a source of both essential (e.g. selenium, iodine) and toxic elements (e.g. arsenic, mercury). Dairy intake was a top predictor in all models, possibly due to its widespread consumption and contribution to both trace mineral intake and competitive absorption dynamics. Fruit and meat also featured prominently, reflecting their importance as contributors to multiple elements. Conversely, foods such as sweets, eggs, legumes and oil had limited predictive value in the multivariate framework, likely reflecting their lower consumption levels or reduced variability in intake within this cohort rather than their nutritional composition. The correlations between food groups are illustrated in Fig. 4.

These multivariate results complement the univariate analyses and further highlight the relevance of considering dietary patterns rather than isolated food–nutrient relationships.

### Multivariate analysis using logistic regression

To further explore the dietary predictors of selenium status, a logistic regression model was constructed using the clinical classification of serum selenium as a binary outcome (adequate (including T3) vs. non-adequate (T1 and T2), adjusting for sex and age. Age was found to be a significant covariate in the model (*p* = 0.01).

The analysis identified associations between several food groups and selenium status (Table 2). Higher seafood intake was significantly linked to increased odds of having adequate selenium levels, while consumption of oil and grains was inversely associated. Consumption levels for legumes and nuts followed positive trends, although these did not reach statistical significance. Nonetheless, these associations are noteworthy given their consistency with the univariate analyses and their potential biological plausibility. Other food groups did not show clear associations with selenium in the adjusted model.

**Table 2:** Logistic regression model predicting adequate serum selenium status based on food group intake, age, and sex

Food group	Odds ratio (OR)	95% CI	*p*-value
Seafood	1.009	1.003–1.015	0.007
Oil	0.947	0.899–0.997	0.018
Grains	0.992	0.984–0.998	0.036
Legumes	1.017	0.999–1.034	0.071
Nuts	1.007	0.995–1.039	0.089

## Discussion

This study provides a comprehensive overview of the relationship between dietary patterns and serum concentrations of essential and toxic trace elements in an adult cohort living in Galicia (north-western Spain). Using a combination of univariate tests, Random Forest models and logistic regression analysis, we identified specific food–element associations that can be interpreted within broader dietary patterns, helping to contextualize the nutritional impact on the trace element profile of the sample population. In parallel with our approach, recent studies have applied machine learning techniques, particularly Random Forest and XGBoost, to explore complex relationships between diet and micronutrient status in different populations and settings. These include models predicting iron, zinc, and copper deficiencies and anaemia risk in children [31], as well as identifying micronutrient intake patterns associated with undiagnosed hypertension in older adults [32] and also comparing machine learning with traditional regression methods to predict serum folate and vitamin B12 levels in healthy individuals [33]. Together, these examples highlight the growing importance of machine learning in nutritional epidemiology and support its use as a complementary tool alongside traditional dietary approaches for enhancing biomarker-based dietary assessment.

Among the essential elements analyzed, selenium clearly stands out with the most consistent associations across all analytical approaches. This result is particularly important given that selenium is the trace element with the most evident deficiency in our study population [19] These findings indicate that circulating selenium is highly responsive to dietary intake and should not be interpreted as evidence of overall nutritional adequacy. Rather, they support the use of selenium as a biomarker of selenium exposure and selenium-specific nutritional status, informing the development of targeted dietary strategies in populations lacking fortification policies.

Previous analyses in this population have identified distinct dietary patterns consistent with a traditional Atlantic diet, characterized by higher consumption of fish, legumes and oils, and lower intake of sweets and processed foods [26]. The associations observed in the present study between specific food groups and serum selenium status align well with these previously described patterns, reinforcing the relevance of dietary combinations rather than isolated foods in shaping selenium status.

Fish consumption emerged as the most relevant positive dietary predictor of selenium levels in our study, consistent with its recognized role as a major dietary source of this element [34–36]. It is also well established that nuts, particularly Brazil nuts, are rich in selenium [37]. However, in the sample cohort, a positive trend in nut consumption was only observed in the logistic regression model and did not reach statistical significance. This probably reflects their relatively limited intake in the dietary patterns of this population, in which fish remains a more prevalent and impactful source of selenium. These findings highlight the need to consider not only the nutrient density of individual foods, but also their actual frequency of consumption and cultural relevance in regional diets. This interpretation is further supported by a recent randomized controlled trial demonstrating that daily intake of Brazil nut butter, providing 55 µg of selenium, significantly improved serum selenium concentrations and selenoP levels in both vegans and omnivores, performing comparably to a standardized selenium supplement [38].

A positive association between selenium and legume intake was also observed, along with a negative association with oils (Table 1). However, legumes are not considered important dietary sources of selenium [36], suggesting that this association may be indirect. One plausible explanation is the existence of an overall healthier dietary pattern: individuals who consume more fish—which is the main dietary contributor to selenium in this cohort—may also consume more legumes and less oil. To explore this hypothesis, we analyzed the correlations between fish intake and the consumption of other food groups (Fig. 4). A positive correlation between consumption of fish and of legumes was observed, but no significant association between consumption of fish and of oil was found. This suggests that the link between selenium and legumes may reflect a shared dietary pattern, while the inverse association with oils could arise from other mechanisms. In fact, excessive lipid intake has been shown to impair intestinal function and nutrient absorption. High-fat diets may alter the intestinal mucosa, disrupt gut microbiota composition and increase oxidative stress [39, 40], all of which could reduce the bioavailability of micronutrients such as selenium. In addition, recent animal studies have demonstrated that selenium supplementation can restore intestinal barrier function and antioxidant defence under conditions of oxidative stress [41]. These findings reinforce the hypothesis that oil intake may influence selenium status through physiological rather than behavioural mechanisms.

In addition to its central role in selenium status, fish consumption also proved to be a key dietary predictor of iodine levels, highlighting its dual contribution to essential trace element intake. This finding is consistent with those of previous studies reporting positive associations between fish consumption and circulating concentrations of both selenium and iodine [42–45]. We also identified a positive association between iodine status and cereal consumption. While cereals are not direct sources of iodine, this relationship likely reflects the use of iodized salt in the manufacturing of industrial bakery products, particularly bread, which is a staple in the Spanish diet. Some processed cereals and breakfast products may also contain added iodized salt, contributing indirectly to iodine intake. In Spain, the use of iodized salt, which is regulated at approximately 60 mg of iodine per kilogram, has been promoted as a public health strategy to ensure adequate iodine intake [46, 47]. Similar strategies have proven successful in other high-income countries, including Denmark, the Netherlands, and Australia, where mandatory or voluntary fortification of bread with iodized salt has contributed substantially to improving population iodine status [48, 49]. These findings highlight how food processing practices and policy-driven interventions can significantly influence micronutrient intake, complementing individual dietary choices. Recent evidence from Germany confirms that use of iodized salt remains the strongest determinant of iodine status at the population level, underscoring the lasting impact of such public health strategies [48–50]. These findings underscore how food processing practices and policy-driven interventions can significantly influence micronutrient intake, complementing individual dietary choices.

For the remaining essential elements, i.e. iron, zinc, copper and manganese, no consistent dietary associations were identified. It should be acknowledged that serum concentrations of zinc, copper and manganese are not considered optimal biomarkers of habitual dietary intake in well-nourished populations, due to tight homeostatic regulation [2]. As a result, circulating levels of these elements tend to remain within relatively narrow ranges and are often insensitive to moderate variations in dietary intake. While some weak or inconsistent associations were observed in the univariate analyses, none of the remaining essential elements showed strong or consistent predictive patterns in the multivariate models. Importantly, serum concentrations of these elements were within normal ranges for all individuals in the cohort, indicating adequate nutritional status across the population. This probably reflects the efficiency of homeostatic mechanisms, particularly intestinal regulation of absorption and excretion, which help maintain serum mineral levels when dietary intake is close to dietary reference values. For instance, zinc homeostasis involves the dynamic regulation of intestinal transporters (ZIP4, ZNT1) and endogenous faecal excretion, making serum zinc relatively insensitive to moderate variability in intake in well-nourished individuals [51]. Similarly, iron absorption is tightly controlled by enterocyte transport mechanisms and hepcidin-dependent signalling pathways that adapt dynamically to iron stores in the body [52]. However, in settings where dietary intake is chronically insufficient, such as in low-income populations with low consumption of animal-source foods, these regulatory mechanisms may no longer be sufficient to maintain adequate serum levels. In such cases, homeostatic control becomes insufficient to compensate for low nutrient intake, and biochemical markers more clearly reflect underlying deficiencies. This has been particularly well documented for iron and zinc, as serum levels of these minerals tend to decline in populations with limited access to meat, fish, or dairy, leading to common outcomes such as anaemia, stunted growth and increased infection risk [53]. These examples highlight how the absence of strong serum–diet associations in well-nourished populations may reflect effective homeostasis, whereas in deficient contexts, dietary patterns become more directly evident in biomarker levels.

Regarding toxic elements, the most consistent associations were observed for mercury and arsenic. In both cases, fish consumption was positively associated with serum levels, in line with numerous previous studies identifying fish, particularly large and long-lived species, as primary sources of exposure to these metals in humans [54–57]. The fact that fish was also a key contributor to optimal selenium and iodine status in our cohort underscores its complex nutritional role as a source of both essential and potentially toxic trace elements. This dual contribution highlights the need to balance the benefits and risks associated with fish intake in dietary recommendations. In the study population, overall exposure to arsenic and mercury remained within the normal reference intervals for non-exposed healthy individuals and did not reach levels associated with adverse health outcomes, even among older adults in whom some bioaccumulation was observed [19]. Importantly, the benefits of fish consumption can be further maximized by prioritizing small and medium-sized species, which have lower levels of mercury and arsenic due to their shorter lifespan and lower trophic level [58, 59]. This dietary approach enables promotion of fish intake as part of public health strategies without compromising toxicological safety.

It should be noted that blood concentrations of certain toxic elements primarily reflect recent exposure rather than long-term intake. In the case of cadmium, circulating levels are considered markers of relatively recent exposure, whereas cumulative or long-term exposure is more accurately assessed using urinary cadmium concentrations [60]. This limitation is even more pronounced for arsenic, for which urinary arsenic is widely recognized as the most appropriate biomarker of chronic exposure [61]. These matrix-specific considerations may partly explain the modest or inconsistent associations observed between dietary intake estimates derived from FFQs and circulating levels of some toxic elements.

In addition to fish, arsenic levels were positively associated with wine and fruit consumption (Table 1). The association with wine may be explained by the historical use of arsenic-based fungicides in vineyards, leading to residual contamination in soils that can persist for decades and be transferred to the final product through the soil–vine–wine pathway. Although arsenic concentrations in wine are generally low and below legal thresholds, previous studies conducted in wine-producing regions have documented measurable levels of arsenic in grapes and wine, especially in soils with a legacy of contamination [62, 63]. On the contrary, the observed positive association between arsenic and fruit consumption was unexpected and should be interpreted with caution. Given the absence of recent evidence identifying fresh fruit as a major contributor to total arsenic exposure in European diets, this finding may instead reflect broader dietary patterns, such as increased fruit consumption among individuals with higher fish intake (Fig. 4).

Several noteworthy negative associations were also observed in our study, particularly between cereal consumption and the levels of arsenic, nickel, chromium and copper. One plausible explanation involves the high content of phytic acid (or phytates) in whole grains, which can chelate divalent and trivalent metal ions, such as As³⁺, Ni²⁺, Cr³⁺ and Cu²⁺, in the gastrointestinal tract, thereby reducing their intestinal absorption. These metals share physicochemical properties such as positive charge and coordination capacity, which facilitate their interaction with the negatively charged phosphate groups of phytic acid. This mechanism is well documented for essential elements such as iron and zinc, particularly in plant-based diets including low consumption of animal-source foods [64]. Emerging evidence further suggests that phytates may also bind toxic metals such as cadmium, lead, chromium and nickel [65]. Although direct human data are limited for some of these toxic elements, our findings support the hypothesis that cereal-rich diets could influence the bioavailability of such elements. This warrants further investigation to determine whether such dietary patterns may offer a protective effect against heavy metal absorption, particularly in settings with environmental exposure.

Other important negative associations included the inverse relationship between dairy intake and arsenic levels, probably mediated by the competitive effect of calcium on intestinal absorption. Recent in vitro digestion studies show that calcium can reduce the bioavailability of arsenic in simulated gastrointestinal conditions, lowering the availability of As³⁺ and As⁵⁺ by approximately 40–70%, depending on the digestive phase (gastric or intestinal) [66]. Likewise, a negative association between wine consumption and serum zinc levels was found. This finding is consistent with evidence from clinical studies showing that chronic alcohol intake can impair intestinal integrity and reduce zinc absorption, leading to lower serum concentrations in individuals with alcohol use disorder [67] as also reported in broader clinical assessments of trace element imbalances in chronic alcohol consumers [68]. Although a similar trend was observed for molybdenum, further research is needed to clarify the mechanisms involved and determine whether this reflects a true malabsorptive effect or broader dietary patterns.

From a multivariate perspective, the Random Forest analysis using all food groups as predictors across the full panel of trace elements suggested that the most influential dietary contributors to the overall mineral profile were fish, dairy products, fruit and vegetables. This finding aligns with recent global studies highlighting these food groups as some of the most micronutrient-dense components of the human diet. In particular, non-starchy vegetables, seafood, dairy and organ meats have consistently been ranked among the top sources of multiple vitamins and minerals, including selenium, iodine, zinc and iron [9, 69, 70]. In parallel the importance of nutrient-rich foods using standardized indices, such as the Nutrient Rich Foods (NRF) score, which systematically classifies foods on the basis of their contribution to essential nutrient intake were emphasized [71]. By contrast, less frequently consumed or lower-density foods, such as legumes, eggs, nuts, sweets and fats, exhibited limited explanatory power for the overall mineral status, with some exceptions like selenium in nuts. These findings further highlight the importance of prioritizing inherently nutrient-dense foods in population diets to address both deficiencies and imbalances in micronutrient profiles.

Among the trace elements analyzed, selenium was the only one for which a logistic regression model was constructed, given its high prevalence of inadequacy in this cohort and its public health relevance [19]. In contrast to other essential elements, for which serum levels were consistently within the adequate range across the population, selenium exhibited sufficient clinical variability to justify a predictive model based on dietary intake. This model identified regular fish consumption as the most consistent dietary predictor of adequate selenium status, reinforcing the well-established role of seafood as a key source of this micronutrient. Notably, individuals with sufficient serum selenium levels reported an average fish intake equivalent to approximately four servings per week, suggesting a realistic dietary threshold that could guide future nutritional recommendations.

While fish was the most consistent contributor, a positive trend was also observed for nut consumption, although statistical significance was not retained in the logistic model. Given the low habitual intake of nuts in this population, this signal (despite the high variability in dietary data) should not be dismissed. Nuts, particularly Brazil nuts, are known to contain high concentrations of selenium, and promotion of nut consumption could serve as a complementary strategy in selenium-deficient groups.

Interestingly, the model also revealed a non-significant but consistent negative association between cereal consumption and selenium status. This trend echoes the inverse associations observed for other divalent and trivalent metal ions (e.g. arsenic, nickel, copper, chromium) and may reflect the inhibitory effect of phytates present in whole grains on mineral absorption. Likewise, a negative trend with intake of fat, particularly oil, aligns with previous hypotheses suggesting that high-fat diets may impair selenium absorption by altering intestinal mucosa or oxidative balance. Although these secondary findings require cautious interpretation, they open interesting avenues for further research and suggest that, in addition to promoting selenium-rich foods, moderation of certain dietary components, such as refined cereals and unhealthy fats, could contribute to optimizing selenium bioavailability.

This study has several strengths that contribute to its scientific and practical value. First, it is based on a well-characterized adult cohort with detailed dietary data and serum trace element concentrations, enabling an integrative analysis of nutritional biomarkers in a real-life population setting. The combination of traditional statistical methods, machine learning techniques and logistic regression models provided complementary perspectives and strengthened the robustness of the associations observed. In particular, the consistent findings regarding selenium reinforce the value of this approach for identifying food-based predictors of micronutrient status. Furthermore, the inclusion of multiple trace elements (both essential and toxic) offers a comprehensive overview of the dietary influences on mineral balance, beyond single-nutrient analyses.

Nonetheless, certain limitations must be acknowledged. The cross-sectional design limits the ability to infer causality, as observed associations may be influenced by reverse causation or residual confounding factors that cannot be fully accounted for in observational studies [72]. Dietary intake was assessed through self-reported food frequency questionnaires, which are inherently subject to recall bias, misreporting and measurement error, particularly for foods consumed irregularly or seasonally [73]. Such uncertainty may attenuate true associations between habitual intake and biomarker concentrations.

In addition, serum concentrations reflect relatively recent exposure rather than long-term nutritional status for several trace elements, and they may be influenced by physiological, metabolic or inflammatory factors unrelated to diet [2, 21]. This limitation is particularly relevant for toxic elements such as cadmium and arsenic, for which blood concentrations primarily reflect recent exposure, while cumulative exposure is more accurately assessed using urinary biomarkers. The lack of arsenic speciation further limits the ability to distinguish between organic and inorganic forms and to identify specific dietary exposure sources. Finally, the relatively low frequency of intake of certain selenium-rich foods (e.g. nuts) in this population may have reduced the statistical power to detect associations with smaller effect sizes, especially in the context of high interindividual variability in dietary reporting.

## Conclusion

This study highlights the value of combining dietary assessment with objective biomarkers to identify critical nutritional gaps and modifiable determinants. In a population with overall adequate trace element status, selenium appears to be the most vulnerable micronutrient, both in terms of public health relevance and predictive dietary modelling. The consistent association with fish intake, supported by logistic regression, underscores the potential to develop simple, food-based strategies to improve selenium status at the population level. While fish remains the most reliable source in this context, the positive trend observed for nuts suggests alternative or complementary dietary options that deserve further exploration. At the same time, the inverse trends linked to cereals and fats indicate the complexity of nutrient interactions and the need to consider bioavailability (not only intake) in nutritional recommendations. Altogether, these findings demonstrate the utility of integrated analytical approaches for mapping nutritional vulnerabilities and generating practical, evidence-informed interventions. They also lay the groundwork for future research into predictive models of micronutrient status and personalized dietary guidance in populations at risk.

## Data Availability

The data needed to replicate the results of the study are available upon reasonable request to the corresponding author and after approval by all the participating institutions.
